# Supplementary material for: SARS‐CoV‐2 nucleocapsid protein phase‐separates with RNA and with human hnRNPs
Source: EMBO J. 2020 Dec 4;39(24):e106478. doi: 10.15252/embj.2020106478 (PMC7737613; doi:10.15252/embj.2020106478)
Supplement: Supplementary file 1 — Appendix [file EMBJ-39-e106478-s001.pdf]

Appendix Figures:

SARS-CoV-2 nucleocapsid protein undergoes phase separation with RNA and partitions with human hnRNPs

Theodora Myrto Perdikari*[1], Anastasia C. Murthy*[2], Veronica H. Ryan*[3], Scott Watters*[4], Mandar T. Naik[4], Nicolas L. Fawzi[4,5#]

[1]Center for Biomedical Engineering
[2]Molecular Biology, Cell Biology & Biochemistry Graduate Program
[3]Neuroscience Graduate Program
[4]Department of Molecular Pharmacology, Physiology, and Biotechnology
[5]Robert J. and Nancy D. Carney Institute for Brain Science
Brown University, Providence, RI, USA

*These authors contributed equally

[#]Correspondence: nicolas_fawzi@brown.edu

Keywords: biomolecular condensates, intrinsically disordered proteins, liquid-liquid phase separation, heterogeneous nuclear ribonucleoproteins, RNA-binding proteins

Table of Contents **Page Number**

Table of Contents	Page Number
Appendix Figure S1	2
Appendix Figure S2	2
Appendix Figure S3	3

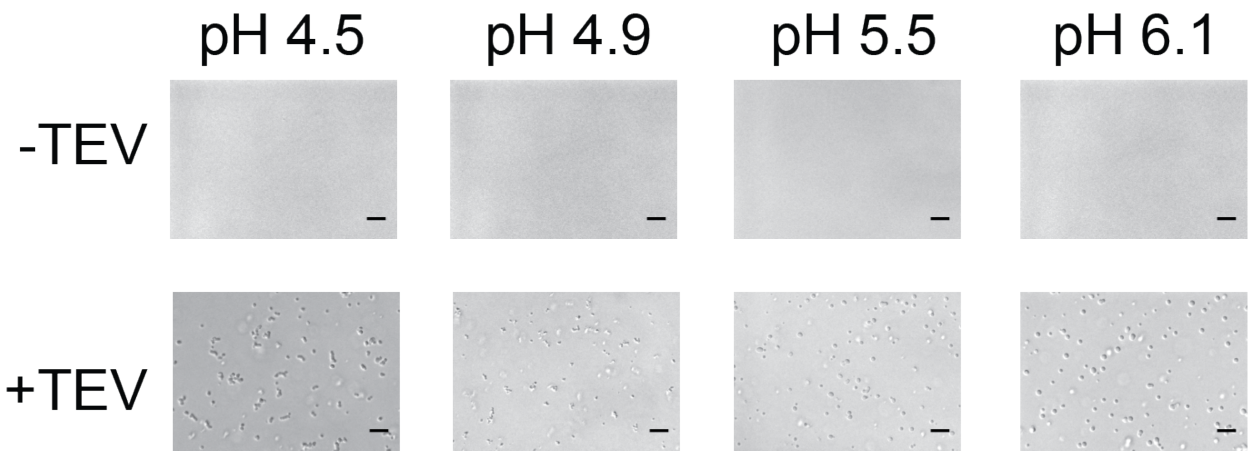

Appendix Figure S1: Low pH conditions induce aggregation of N in the presence of RNA. A) DIC micrographs of 50 μM N in varying pH conditions without and with TEV protease to cleave the MBP tag to initiate phase separation. At lower pH conditions, droplets appear to be non-spherical, consistent with less fluid behavior. Scale bars represent 50 μm.

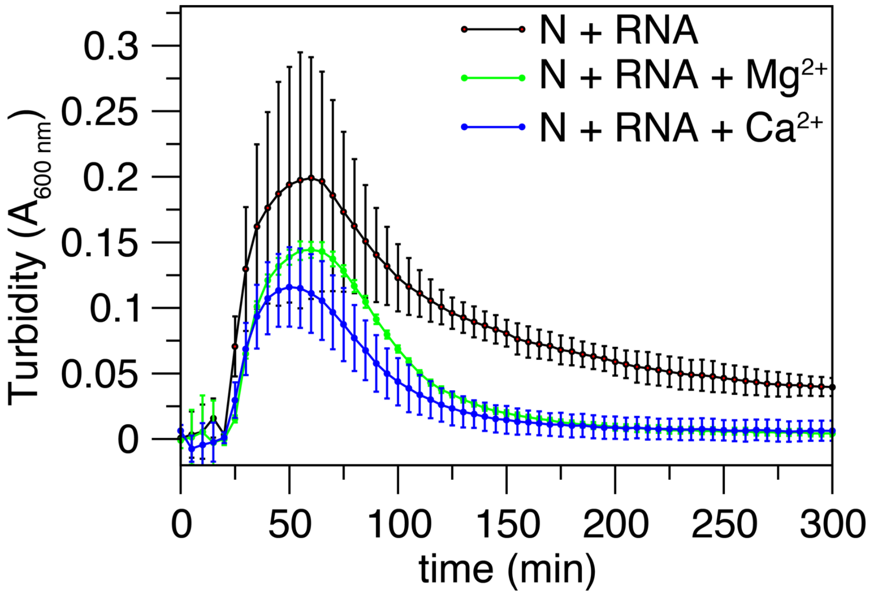

Appendix Figure S2: Divalent metal salts do not substantially alter N LLPS. Addition of 2 mM $MgCl_2$ or $CaCl_2$ does not alter LLPS of 50 μM MBP-N in the presence of 0.5 mg/mL RNA in 50 mM Tris, 70 mM NaCl, pH 7.4 at room temperature. Error bars are standard deviation of three replicates.

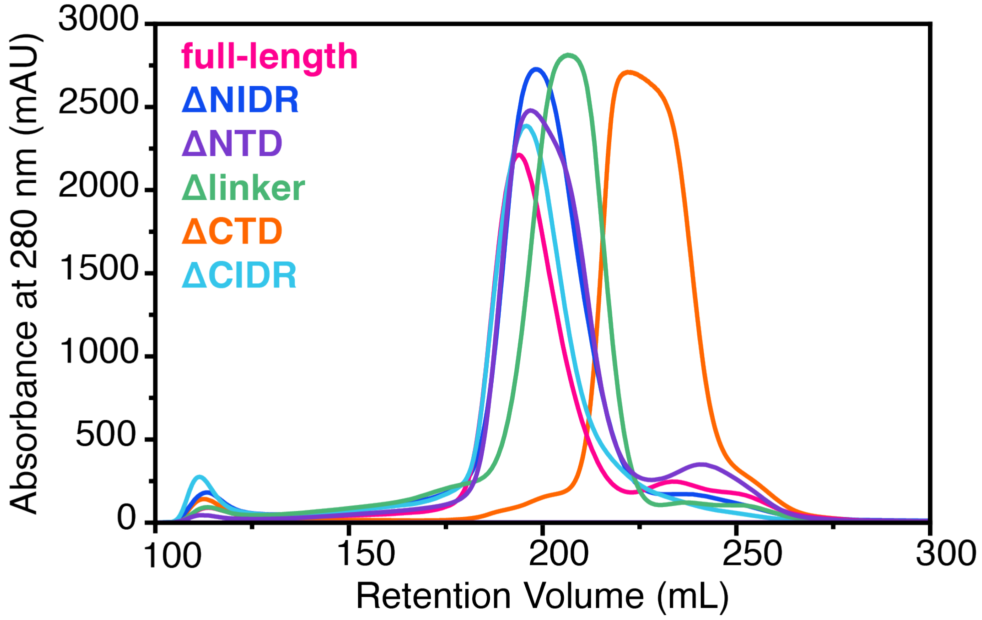

Appendix Figure S3: Preparative scale gel filtration of N and N domain deletion variants. Elution times for samples injected on a HiLoad 26/600 Superdex 200 pg column equilibrated in 20 mM Tris, 1.0 M NaCl, pH 8.0.
