# Supplementary material for: SARS‐CoV‐2 nucleocapsid protein phase‐separates with RNA and with human hnRNPs
Source: EMBO J. 2020 Dec 4;39(24):e106478. doi: 10.15252/embj.2020106478 (PMC7737613; doi:10.15252/embj.2020106478)
Supplement: Supplementary file 3 — Source Data for Figure 7 [file EMBJ-39-e106478-s002.pdf]

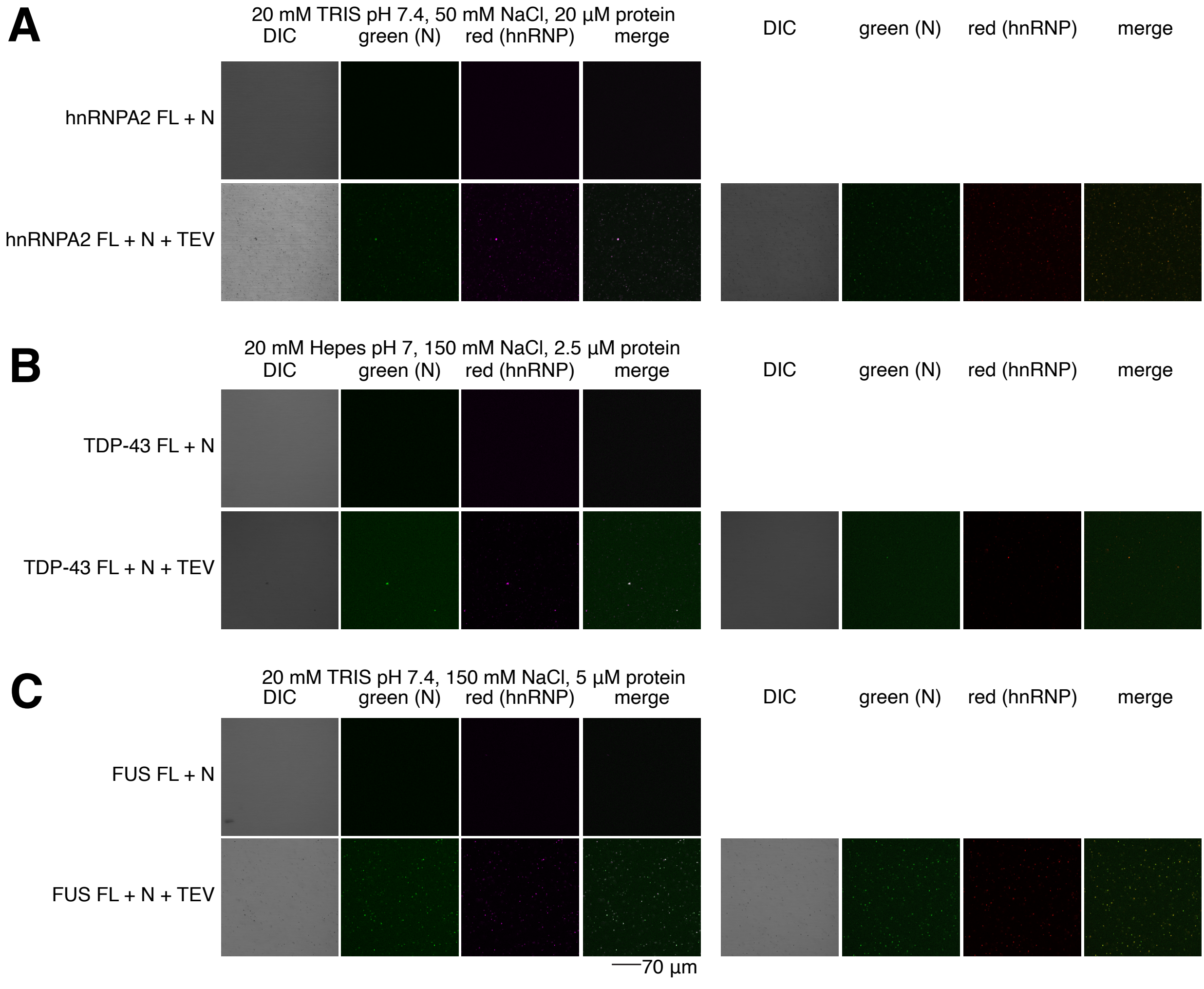
A
20 mM TRIS pH 7.4, 50 mM NaCl, 20 µM protein
DIC
green (N)
red (hnRNP)
merge
DIC
green (N)
red (hnRNP)
merge
hnRNPA2 FL + N
hnRNPA2 FL + N + TEV
B
20 mM Hepes pH 7, 150 mM NaCl, 2.5 µM protein
DIC
green (N)
red (hnRNP)
merge
DIC
green (N)
red (hnRNP)
merge
TDP-43 FL + N
TDP-43 FL + N + TEV
C
20 mM TRIS pH 7.4, 150 mM NaCl, 5 µM protein
DIC
green (N)
red (hnRNP)
merge
DIC
green (N)
red (hnRNP)
merge
FUS FL + N
FUS FL + N + TEV
70 µm
